# Efficacy of endoscopic gallbladder drainage in patients with acute cholecystitis

**DOI:** 10.1186/s12893-022-01676-y

**Published:** 2022-06-11

**Authors:** Anri Kaneta, Hirotaka Sasada, Takuma Matsumoto, Tsuyoshi Sakai, Shuichi Sato, Takashi Hara

**Affiliations:** Department of Surgery, Kensei Hospital, 2 Ogimachi, Hirosaki, Aomori 036-8511 Japan

**Keywords:** Acute cholecystitis, Gallbladder drainage, Endoscopic trans-papillary gallbladder drainage, Percutaneous transhepatic gallbladder drainage, Laparoscopic cholecystectomy, Subtotal cholecystectomy

## Abstract

**Background:**

Early cholecystectomy is recommended for patients with acute cholecystitis. However, emergency surgery may not be indicated due to complications and disease severity. Patients requiring drainage are usually treated with percutaneous transhepatic gallbladder drainage (PTGBD), whereas patients with biliary duct stones undergo endoscopic stones removal followed by endoscopic gallbladder drainage (EGBD). Herein, we investigated the efficacy of EGBD in patients with acute cholecystitis.

**Methods:**

Overall, 101 patients receiving laparoscopic cholecystectomy between September 2019 and September 2020 in our department were retrospectively analyzed.

**Results:**

The patients (n = 101) were divided into three groups: control group that did not undergo drainage (n = 68), a group that underwent EGBD (n = 7), and a group that underwent PTGBD (n = 26). Median surgery time was 107, 166, and 143 min, respectively. Control group had a significantly shorter surgery time, whereas it did not significantly differ between EGBD and PTGBD groups. The median amount of bleeding was 5 g, 7 g, and 7.5 g, respectively, and control group had significantly less bleeding than the drainage group. We further divided patients into the following subgroups: patients requiring a 5 mm clip to ligate the cystic duct, patients requiring a 10 mm clip due to the thickness of the cystic duct, patients requiring an automatic suturing device, and patients undergoing subtotal cholecystectomy due to impossible cystic duct ligation. There was no significant difference between EGBD and PTGBD regarding the clip used or the need for an automatic suturing device and subtotal cholecystectomy.

**Conclusions:**

There was no significant difference between EGBD and PTGBD groups regarding surgery time or bleeding amount when surgery was performed after gallbladder drainage for acute cholecystitis. Therefore, EGBD was considered a useful preoperative drainage method requiring no drainage bag.

## Background

Early cholecystectomy is a standard therapy for acute cholecystitis (AC) [1, 2]. However, early surgical intervention may result in increased morbidity and mortality in the elderly, patients with multiple comorbidities, or those with advanced cholecystitis [3]. According to Tokyo guidelines 2018, early gallbladder drainage is should be considered for patients with severe local inflammation and/or severe (grade III) AC [4].

Percutaneous transhepatic gallbladder drainage (PTGBD) is a widely performed and established method for gallbladder drainage. However, PTGBD is generally prohibited in patients with a breeding tendency, massive ascites, and anatomically inaccessible gallbladders. In addition, PTGBD is associated with adverse events, including bleeding, and catheter dislodgement. There are several reports on the usefulness and safety of endoscopic gallbladder drainage (EGBD), including endoscopic nasogallbladder drainage (ENGBD) and endoscopic gallbladder stenting, and endoscopic ultrasound-guided gallbladder drainage (EUS-GBD) in patients with AC [5–7]. However, there have been few studies comparing the feasibility of laparoscopic cholecystectomy (LC) for AC after EGBD and LC after PTGBD [8, 9]. The objective of this study was to evaluate the feasibility of LC after EGBD compared with PTGBD.

## Methods

### Study population

**Fig. 1:**
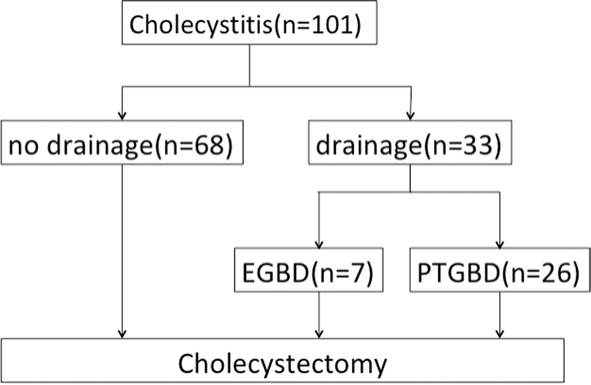
Flow of 101 patients who underwent cholecystectomy for acute cholecystitis

Overall, 101 patients who underwent LC for AC between September 2019 and September 2020 in our department were retrospectively analyzed. Of these, 33 patients who underwent LC for AC after gallbladder drainage were included in the analysis (Fig. 1). This retrospective study was approved by the Medical Ethics Committee of Kensei Hospital (no. 2021-03) and performed following the ethical guidelines for clinical studies.

### Treatment

In our institution, urgent or semi-urgent LC was performed for AC patients tolerant for surgery. If surgery was unsuitable, AC patients were treated in a gastroenterology department. In addition to antibiotic treatment, gallbladder drainage was performed depending on disease course and severity. In most cases, PTGBD was selected as the drainage method. EGBD was selected for patients suspected of choledocholithiasis, bleeding tendency, and dementia with a risk of drainage tube self-removal.

### PTGBD

PTGBD was guided by ultrasound. After an 18-gauge needle was inserted into the gallbladder, a guidewire was coiled into the gallbladder. And then, a pigtail catheter was placed using a guidewire under fluoroscopy.

### EGBD

**Fig. 2:**
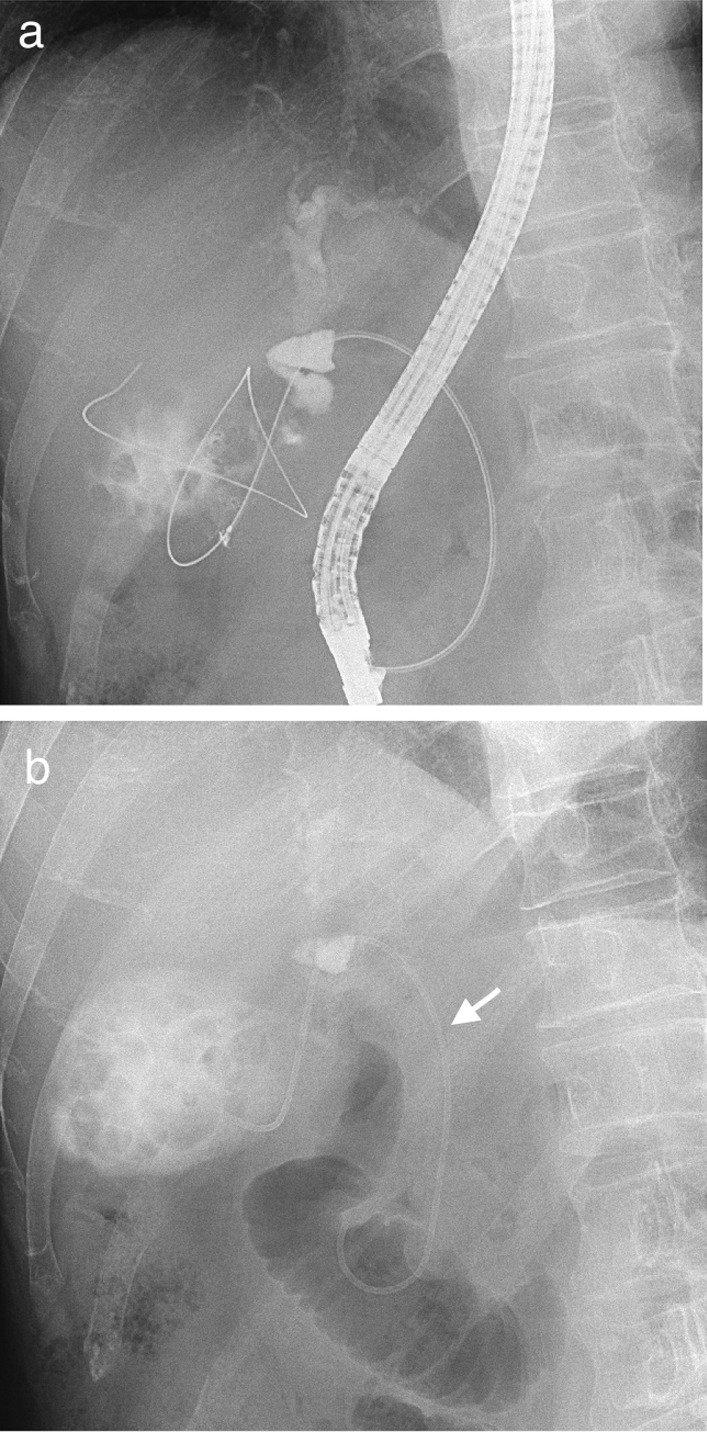
Endoscopic retrograde cholangiopancreatography (ERCP) catheter was inserted into the cystic duct (**a**). The arrowhead shows that the 5-French IYO-stent™ was inserted into the gallbladder for drainage (**b**)

The term EGBD generally includes ENGBD and EUS-GBD, but these are not performed in our institution. In the text, EGBD means endoscopic trans-papillary gallbladder drainage. Following endoscopic retrograde cholangiopancreatography (ERCP), a 0.035-inch Radifocus guidewire (Terumo, Tokyo, Japan) was advanced into the cystic duct and subsequently into the gallbladder. A 5-French IYO-stent™ (32 cm, Gadelius Medical K.K., Tokyo, Japan) was inserted into the gallbladder along the guidewire (Fig. 2).

### Surgery after drainage

Because most studies determined that a short interval between PTGBD and LC can increase the intraoperative difficulty [10–12], LC was basically performed at least 2 months after drainage so that edema and inflammation around the gallbladder subsided. All EGBD tubes were removed before LC.

### Patient variables

The characteristics of EGBD and PTGBD patients before gallbladder drainage were compared. Surgical results in patients with and without gallbladder drainage were compared. In addition, surgical results in EGBD and PTGBD patients were compared. The severity of AC was determined by the Tokyo guideline criteria [4].

### Statistical analysis

Categorical variables were compared using the chi-square test and Fisher’s exact test, and continuous variables were compared using Mann–Whitney U-test. A *P* < 0.05 was considered significant for all tests. The statistical analysis was performed with js-STAR XR release 1.1.3j.

## Results

Patient status before drainage is summarized in Table 1. There were no significant differences in age, sex, anticoagulant therapy, common bile duct stone, dementia, and severity of AC between the two groups. The time to operation day was significantly shorter in PTGBD group. In PTGBD group, there were 4 cases of the drainage tube obstruction and 5 cases of dislodgment, whereas in EGBD group, there was no stent trouble. Furthermore, in PTGBD group, there were 6 patients who were forced to continue hospitalization until surgery due to the inability to manage the drainage tube at home, but not in EGBD group. There was no significant difference, but five PTGBD patients had severe Tokyo grade III AC whereas there were no grade III patients in EGBD group.

**Table 1:** Characteristics of EGBD and PTGBD patients before gallbladder drainage

Characteristics	EGBD (n = 7)	PTGBD (n = 26)	*p* value
Age	70 (63–85)	71 (56–88)	0.7
Sex (male/female)	4/3	14/12	1.0
Anticoagulant therapy	2 (28%)	8 (31%)	1.0
Common bile duct stone	1 (14%)	3 (11%)	1.0
Dementia	0 (0%)	4 (15%)	0.55
Adverse events			
Tube obstruction	0 (0%)	4 (15%)	0.55
Tube dislodgment	0 (0%)	5 (19%)	0.56
Continued hospitalization until surgery	0 (0%)	6 (23%)	0.30
Time to operation (day)	96 (26–124)	66 (32–122)	0.03
Grade*			
I	3 (43%)	9 (35%)	0.71
II	4 (57%)	12 (46%)	
III	0 (0%)	5 (19%)	

*Classified by the Tokyo guidelines

Data are presented as median (range) or number

Table 2 shows the background in which EGBD was selected. EGBD was performed when common bile duct (CBD) stone was suspected by laboratory data, and when PTGBD was not suitable due to patient requirements or physical disability. As a result of ERCP, only one case actually had CBD stones. Endoscopic sphincterotomy (EST) was performed in all case.

**Table 2:** Characteristics of EGBD patients

No.	Age (yrs)	Gender	CBD stone	EST	Dementia	Other diseases	Reasons for choosing EGBD
1	75	F	No	Yes	No	No	Choledocholithiasis was suspected by laboratory data
2	84	F	No	Yes	No	No	The patient requested.(She felt uneasy about drain management)
3	70	M	No	Yes	No	No	Choledocholithiasis was suspected by laboratory data
4	63	M	No	Yes	No	No	The patient requested.(The drain may have hindered his work)
5	65	M	No	Yes	No	No	The gallbladder was not visualized by ultrasound
6	85	F	No	Yes	No	Cerebral hemorrhage (sequelae)	Drain management may have been difficult due to physical disability
7	67	M	Yes	Yes	No	Cerebral hemorrhage (sequelae)	Drain management may have been difficult due to physical disability

Surgical results in patients with and without gallbladder drainage are shown in Table 3. There are significant differences in the variables as follows; surgery time, blood loss, cystic duct closure, and hospital stay. In all of these, non-drainage group had a better result, but only the non-drainage group had postoperative complications.

**Table 3:** Surgical results in patients with and without drainage

	No drainage(n = 68)	Drainage(n = 33)	*P* value
Surgery time (min)	107 (51–304)	148 (75–299)	0.0028
Blood loss (ml)	5 (0–201)	7 (1–202)	0.0085
Conversion to open surgery	0	0	
Cystic duct closure			
5 mm clip	48	9	0.00018
12 mm clip	5	3	
Automatic suturing device	11	16	
Subtotal cholecystectomy	4	5	
Postoperative complication Clavian–Dindo criteria			
No complication	64	33	0.59
Grade II	2	0	
Grade IIIa	2	0	
Postoperative stay(day)	4 (1–17)	7 (2–17)	0.0019

Data are presented as median (range) or number

Intra- and postoperative factors in EGBD and PTGBD groups are summarized in Table 4. The median surgery times were 166 min (range, 76–299) for EGBD and 143 min (range, 75–264) for PTGBD (*P* = 0.4). In both groups, there was no conversion to open surgery.

**Table 4:** Surgical result in EGBD and PTGBD patients

	EGBD	PTGBD	*P* value
Surgery time (min)	166 (76–299)	143 (75–264)	0.4
Blood loss (ml)	7 (2–12)	7.5 (1–202)	0.45
Conversion to open surgery	0	0	–
Cystic duct closure			
5 mm clip	1	8	0.69
12 mm clip	0	3	
Automatic suturing device	5	11	
Subtotal cholecystectomy	1	4	
Postoperative complication	0	0	–
Postoperative stay (day)	6 (3–9)	7 (2–17)	0.28

Data are presented as median (range) or number

If the critical view of safety could not be established, Fundus first technique was performed. Furthermore, if it was difficult to identify the cystic duct, laparoscopic subtotal cholecystectomy was performed. There was no significant difference between the groups in cystic duct closure, but ligation with a 5-mm clip was difficult in EGBD group, and automatic suturing devices tended to be used more often. There were no postoperative complications in both groups. There was no significant difference in postoperative hospital stay between the two groups.

## Discussion

According to 2018 Tokyo guidelines, the first surgical treatment of choice for mild or moderate AC is LC. If it is decided that the patient cannot withstand surgery, conservative treatment and biliary drainage should be considered [4]. In drainage group, there were only 5 patients with grade III AC. It is possible that the drainage group included cases in which surgery should be selected as the initial treatment. In our institution, urgent surgery for mild and moderate AC was sometimes not selected due to patient-side factors such as anticoagulant therapy and comorbidity, and institutional factors such as a shortage of anesthesiologists. After drainage, inflammation may increase the difficulty of cholecystectomy. The optimal timing of cholecystectomy after drainage is still without consensus, but there are some reports that delayed surgery after drainage can be performed more safely than early surgery [10–13]. Then, we required most patients to undergo LC at least 2 months after drainage. In drainage group, although surgery time, blood loss, and hospital stay increased, postoperative complications did not increase, demonstrating the adequacy of the treatment strategy (Table 3).

PTGBD is a frequently performed and established method for gallbladder drainage. A previous systematic review showed that the technical success rate of PTGBD was 98% [14]. However, we speculate that the external tube might decrease the quality of life (QOL) while awaiting surgery due to postprocedural pain and discomfort. Additional associated risks include catheter dislodgment, bile leakage, bleeding, and pneumothorax [15]. On the other hand, EGBD is a complex procedure with a reported success rate of 77–91% [9, 16–21]. Failure of EGBD was mostly attributable to the inability to detect the cystic duct and insert the guidewire or the stent due to an obstruction caused by severe inflammation and gallstones within the duct [7]. Therefore, EGBD requires an expert endoscopist. EGBD had a similar technical success rate to PTGBD but seems to be safer because it has lower complication rate than PTGBD, according to a meta-analysis [22]. It has also been reported that EGBD is superior to PTGBD in the patient’s QOL and hospitalization period [9, 21, 23]. We did not evaluate QOL after the drainage. But as shown in Table 1, the drainage tube trouble often occurred, and there were 6 patients who were forced to continue hospitalization until surgery in PTGBD group. It is considered that the time to operation was significantly shortened due to discomfort and trouble with the drainage tube. In most cases, the ultimate goal of treating AC is safe cholecystectomy. The optimal timing of LC after drainage is still without consensus, we required most patients to undergo LC at least 2 month after drainage based on past reports. It is important to have a sufficient waiting period before surgery without long-term hospitalization or frequent visits. Although the efficacy of EGBD is gradually being established, there are few reports on its effect on surgery. There are concerns that surgery after EGBD might be more difficult because inflammation around the cystic duct and cannulation of the drainage tube interfere with dissection in Calot’s triangle. In this patient series, only in one case in EGBD group, a ligation with a 5-mm clip was possible; thus, the cystic duct may tend to thicken after EGBD. However, there was no significant difference between EGBD and PTGBD in cystic duct closure. Cannulation of the drainage tube may not affect dissection in Calot’s triangle so much. And then EGBD did not increase the difficulty of surgery compared with PTGBD. Surgery time and blood loss were equivalent. The postoperative complication and hospital stay were also equivalent (Table 4). Therefore, EGBD was considered useful as a preoperative drainage method. This study found that LC after drainage was safe and feasible, but more difficult. It was also found that PTGBD impaired the patient’s QOL and extended hospital stay. In the future, it will be necessary to carefully judge whether drainage is appropriate and whether endoscopic drainage is possible in order to provide safe and low-burden treatment for AC.

The limitation of this study was its retrospective analysis, a small number of patients, and investigation in a single institution. In order to further explore the actual feasibility of LC after EGBD, it needs to be investigated by prospective studies.

## Conclusions

EGBD could be a safe and effective alternative treatment to PTGBD for patients with AC who are unsuitable for emergency cholecystectomy. This study showed that LC was performed successfully and safely after either EGBD or PTGBD. The feasibility of LC after EGBD was comparable to LC after PTGBD. However, based on the limits of the current study, large sample, multi-center studies are still needed.

## Data Availability

The data and materials used and/or analyzed during the current study are available from the corresponding author on reasonable request.

## Abbreviations

AC: Acute cholecystitis
EGBD: Endoscopic gallbladder drainage
LC: Laparoscopic cholecystectomy
PTGBD: Percutaneous transhepatic gallbladder drainage
CBD: Common bile duct
EST: Endoscopic sphincterotomy
